# A colorimetric and fluorescent signaling probe for assaying Pd^2+^ in practical samples†

**DOI:** 10.1039/d3ra05549c

**Published:** 2023-11-01

**Authors:** Myung Gil Choi, Juyoung Han, Sangdoo Ahn, Suk-Kyu Chang

**Affiliations:** a Department of Chemistry, Chung-Ang University Seoul 06974 Republic of Korea sangdoo@cau.ac.kr skchang@cau.ac.kr +82 2 825 4736 +82 2 820 5199

## Abstract

We developed an optical signaling probe to detect Pd^2+^ ions in Pd-containing catalyst and drug candidate. The Pd^2+^ signaling probe (Res-DT) was readily prepared by reacting the versatile fluorochrome resorufin with phenyl chlorodithioformate. In a phosphate-buffered saline solution (pH 7.4) containing sodium dodecyl sulfate (SDS) as a signal-boosting surfactant, Res-DT exhibited a pronounced colorimetric response with a chromogenic yellow to magenta shift, leading to a substantial increase in the fluorescence intensity. The Pd^2+^ signaling performance of Res-DT was attributed to the Pd^2+^-promoted hydrolysis of the dithioate moiety. The probe displayed high selectivity toward Pd^2+^ ions and remained unaffected by commonly encountered coexisting components. Moreover, the detection limit of Res-DT for Pd^2+^ ions was 10 nM, and the signaling was achieved within 7 min. Furthermore, to demonstrate the real-world applicability of Res-DT, a Pd^2+^ assay was performed in Pd-containing catalyst and drug candidate using an office scanner as an easily accessible measurement device. Our results highlight the prospects of Res-DT as a tool to detect Pd^2+^ ions in various practical samples, with potential applications in catalysis, medicine, and environmental science.

## Abstract 2

A colorimetric and fluorescent signaling probe was developed for Pd^2+^ assay in Pd-containing catalyst and drug candidate, with a rapid response and high selectivity.

## Graphical abstract

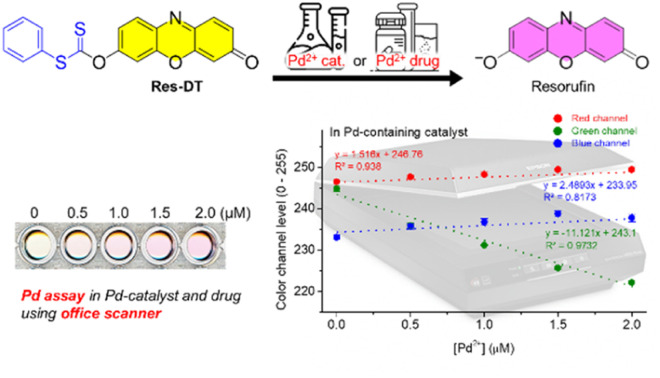

## 1. Introduction

Palladium (Pd) is a versatile and valuable Pt-group metal with diverse practical applications in industrial arenas such as dentistry, electronics, chemical synthesis, groundwater treatment, and exhaust gas treatment.^1,2^ Additionally, it is employed in therapeutics owing to its antiviral, antifungal, antimicrobial, anticancer, and cardioprotective properties.^3^ Furthermore, it is a vital component of fuel cells that generate energy through chemical reactions involving hydrogen and oxygen.^4^

Pd-based catalysts are essential for producing myriad fine chemicals that are used to manufacture pharmaceuticals and agricultural products.^5^ In particular, several Pd-catalyzed organic reactions, including the Mizoroki–Heck reaction,^6^ Suzuki–Miyaura reaction,^7^ Sonogashira–Hagihara reaction,^8^ Buchwald–Hartwig amination,^9^ carbonylation,^10^ and cyanation,^11^ are performed in pharmaceutical preparations.^12^ Moreover, Pd complexes can be used as anticancer agents owing to their similar chemical and physical properties to those of the widely employed Pt complexes.^13^ However, the inherent toxicity of Pd has made Pd contamination a matter of considerable concern.^14^ In particular, Pd species can bind to important biological materials, such as amino acids, DNA, RNA, and proteins,^15,16^ thereby disrupting cellular processes and leading to severe health problems including weight loss, muscle weakness, seizures, and heart disease.^17^ Consequently, the determination of residual Pd in commercially available drug chemicals, agricultural products, and foods is crucial.

Various conventional analytical techniques have been employed to detect Pd in different analytes using sophisticated, specialized analytical instruments.^18^ However, these techniques typically require complex sample preparation protocols, stringent experimental conditions, and highly skilled operators.^19^ In contrast, colorimetric or fluorescent chemosensors and reaction-based probes hold greater promise for selective and sensitive metal ion/anion detection, including Pd. These methods offer straightforward operability, high sensitivity, no need for heavy instruments, and widespread applicability.^20^

To meet the increasing need for simpler and more convenient Pd analysis methods, several colorimetry or fluorescence-based chemosensors and reaction-based probes have been developed.^19,21^ Pd signaling sensors have been obtained using diverse ligands including pyridine-2,6-dicarboxamide,^22^ 2-chloroethyl methyl sulfide,^23^ purine derivative,^24^ and 2-picolylamine.^25^ Additionally, several Pd-selective signaling probes have been developed by leveraging their Pd-selective reactions, signal-accumulating ability, and ease of design.^26^ For instance, deallylation of allyl carbamates,^27^ allyl carbonates,^28^ allyl ethers,^29^ and allyl ester,^30^ and depropargylation of propargyl ethers^31^ and propargyl carbamates^32^ have been extensively performed to design Pd signaling probes. In addition, probes that detect Pd *via* metal-induced organic transformations have been prepared using the Claisen rearrangement,^33^ dimerization,^34^ and oxidative cyclization reactions.^35^ Furthermore, several Pd signaling probes that exploit the hydrolysis of thiocarbamate and hydrazones of resorufin and rhodamine fluorochromes have been engineered.^36^ There have also been various studies exploring Pd^2+^ sensing probes, each contributing valuable insights into their development and applications.^37^ Representative optical Pd specific reaction-based probes have been summarized in Table S1 (ESI).†

In this study, we introduce a novel dual-mode probe (Res-DT) designed for the highly sensitive detection of Pd^2+^ ions through both colorimetric and fluorescent responses, facilitated by the hydrolysis of dithioate-modified resorufin. The probe ensures rapid, convenient, and naked-eye detectable responses, obviating the need for complex instrumentation. Res-DT demonstrates efficiency in swiftly and precisely assaying residual Pd^2+^ in a Pd-containing catalyst and a Pd-containing drug candidate noted for its antimicrobial and anticancer activities.

## 2. Experimental

### 2.1 Synthesis of Res-DT

Resorufin dithioate (Res-DT) was synthesized using a previously reported method with slight modifications.^38^ In a 100 mL round bottom flask, resorufin (0.43 g, 2.0 mmol) was dissolved in 30 mL of *N*,*N*-dimethylformamide (DMF). The solution was then mixed with triethylamine (TEA; 0.56 mL, 4.0 mmol) and stirred for 30 min at room temperature. Phenyl chlorodithioformate (0.45 mL, 3.0 mmol) was then added carefully to the solution, and the reaction was allowed to continue for 12 h with constant stirring. DMF was then removed by passing air over the system, and the remaining solid was dissolved in dichloromethane (50 mL). The resulting solution was washed with distilled water and brine and then evaporated. The obtained residue was purified by column chromatography (CH_2_Cl_2_ : CH_3_OH = 49 : 1, v/v). Res-DT. 0.52 g, 71% yield as a vermillion-colored powder. ^1^H NMR (600 MHz, CDCl_3_) *δ* 7.81 (dt, *J* = 8.9, 1.2 Hz, 1H), 7.64–7.59 (m, 2H), 7.54–7.46 (m, 3H), 7.42 (d, *J* = 9.8 Hz, 1H), 7.13–7.10 (m, 2H), 6.86 (dd, *J* = 9.8, 2.0 Hz, 1H), 6.32 (d, *J* = 2.0 Hz, 1H); ^13^C NMR (150 MHz, CDCl_3_): *δ* 212.4, 186.2, 156.6, 149.1, 148.7, 144.4, 135.3, 135.2, 134.8, 131.8, 131.2, 130.7, 129.8, 129.7, 119.9, 110.6, 107.3; HRMS (EI^+^, *m*/*z*): calcd. for C_19_H_11_NO_3_S_2_^+^ [M]^+^: 365.0180, found 365.0179.

### 2.2 Stock solution preparation

A stock solution containing probe Res-DT (0.5 mM) was made by dissolving Res-DT in dimethyl sulfoxide (DMSO). Pd-containing solutions (5.0 mM) were prepared by dissolving Pd(OAc)_2_, PdCl_2_, and Pd(PPh_3_)_4_ in DMSO, and K_2_PdCl_6_ in deionized (DI) water. Solutions of metal ions and anions (5.0 mM) were prepared using metal perchlorate salts and sodium salts of the anions, respectively, in DI water. Oxidant solutions (5.0 mM), including H_2_O_2_, HOCl, peracetic acid, O_2_^−^, perborate, percarbonate, and ammonium persulfate, were prepared and standardized as described previously.^39^

### 2.3 Investigation of Pd^2+^ signaling with Res-DT

We investigated the Pd^2+^ sensing behavior of Res-DT in a pH 7.4 phosphate-buffered saline (PBS) solution, using 2% DMSO as a solubilizer. To that end, the analyte stock solution (15 μL, 5.0 mM) was first added to a sample tube and diluted with DI water (2.34 mL) and a predetermined amount of DMSO. Then, a PBS solution (0.30 mL, 100 mM) and SDS (0.30 mL, 100 mM) were added to the mixture, followed by Res-DT (30 μL, 0.50 mM). The final concentrations of Res-DT, the analyte, PBS, and SDS were 5.0 μM, 25 μM, 10.0 mM, and 10.0 mM respectively. Error bars were determined based on the standard deviation derived from three sets of experiments.

### 2.4 Mechanism study of Pd^2+^ signaling

In a 100 mL round bottom flask, Res-DT (37 mg, 100 μmol) was dissolved in CH_3_CN (20 mL). The resulting solution was then mixed with palladium acetate (56 mg, 250 μmol). Upon verifying the completion of the reaction by thin-layer chromatography, the precipitate was collected using a centrifuge (4000 rpm) and washed with CH_3_CN. The precipitate was dried and analyzed using field-emission scanning electron microscopy (FE-SEM) equipped with energy-dispersive X-ray spectroscopy (EDX). The remaining solution was evaporated under reduced pressure and the residue was purified by column chromatography. The purified product of the Pd^2+^ signaling of Res-DT was scrutinized by ^1^H NMR and mass spectrometry measurements.

### 2.5 Office scanner-based determination of Pd^2+^ concentration in Pd-containing catalyst and drug candidate

To determine the Pd^2+^ concentration in Pd-containing catalyst and drug candidate, five samples with varying Pd^2+^ levels were tested. These samples were prepared by mixing a predetermined amount of the Pd-containing catalyst or drug (ranging from 0 to 6 μL; 1.0 mM), DI water (2.34 mL), DMSO (15 μL), PBS (0.30 mL, 0.10 M), SDS (0.30 mL, 0.10 M), and Res-DT (30 μL, 0.50 mM) in a sample vial. The resulting solutions (0.30 mL) were then individually transferred into a 96-well plate. A calibration plot of the color channel levels (RGB) against the Pd species concentration was constructed thereafter using an office scanner (V550, Epson) in transmittance mode.

## 3. Results and discussion

**Scheme 1:**
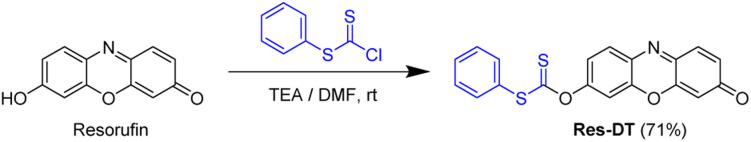
Preparation of the Pd^2+^ signaling probe (Res-DT).

Sulfur-based chemosignaling probes have been extensively used to detect thiophilic metal ions and common oxidants through the desulfurization and oxidative hydrolysis reactions.^38,40^ In the present study, the dithioate moiety, which is prone to desulfurization-induced hydrolysis in the presence of hypochlorite ions,^38^ was targeted in this study to develop a colorimetric, fluorescent signaling probe for analyzing Pd^2+^ in catalysts and drugs. The undesired response toward hypochlorite ions can be readily suppressed using hypochlorite-scavenging DMSO.^41^ Based on this rationale, a simple but optically vibrant dithioate-based Pd^2+^ signaling probe, called Res-DT, was developed. Probe Res-DT was synthesized by reacting resorufin with phenyl chlorodithioformate (Scheme 1) and then characterized by NMR spectroscopy and mass spectrometry.

First, the Pd^2+^ signaling condition of Res-DT was optimized by measuring the changes in absorbance at 572 nm. Preliminary results indicated that Res-DT exhibited moderate Pd^2+^ signaling activity in pH 7.4 PBS with 2% DMSO which acted both as a solubilizer and a scavenger for potential hypochlorite interferants (Fig. S1, ESI†). However, the signaling performance was relatively sluggish for practical applications, given that the reaction was incomplete even after 30 min. Therefore, to improve the reaction rate of Res-DT for Pd^2+^ ions, three types of surfactants were employed: anionic (SDS), cationic (cetyltrimethylammonium bromide (CTAB)), and nonionic (Tween 20). According to the results (Fig. S2a, ESI†), the Pd^2+^ signaling behavior of Res-DT was substantially improved upon using SDS (10.0 mM). The other surfactant solutions (CTAB and Tween 20) also enhanced the Pd^2+^ signaling speed of Res-DT (Fig. S2b and c, ESI†). However, Res-DT was slightly hydrolyzed when the CTAB solution (1.0 mM) was used, and undesirable precipitates were formed when the Tween 20-containing solution (0.09 mM) was employed (Fig. S3, ESI†). Therefore, subsequent Pd^2+^ signaling experiments with Res-DT were performed using the SDS surfactant solution as a signal booster.

**Fig. 1:**
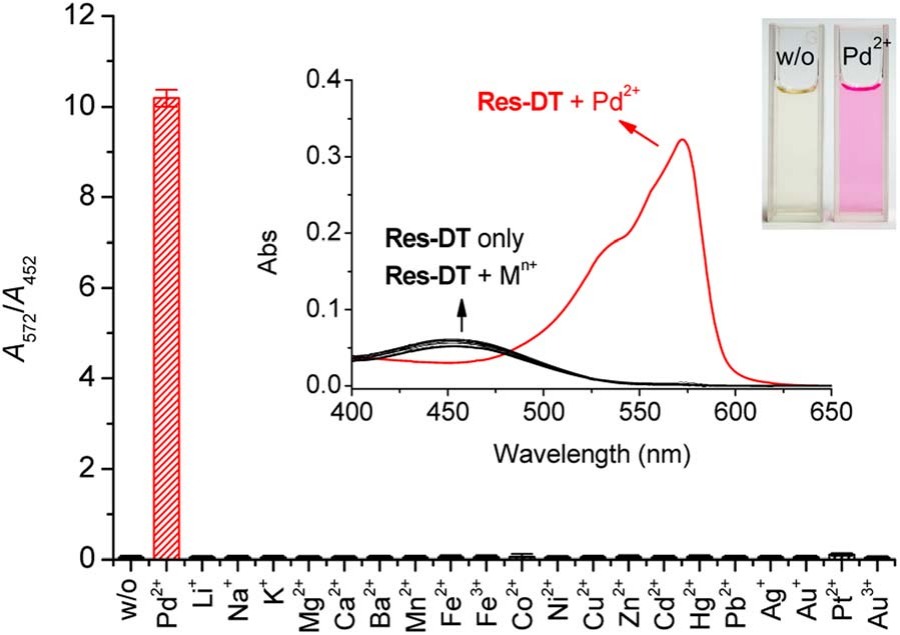
Changes in the absorbance ratio of Res-DT (*A*_572_/*A*_452_) with the incorporation of common metal ions. Inset: UV-vis spectra and naked-eye photographs of Res-DT. [Res-DT] = 5.0 μM, [Pd^2+^] = [M^*n*+^] = 25 μM, [SDS] = 10.0 mM, [PBS pH 7.4] = 10.0 mM in an aqueous solution containing 2% (v/v) DMSO.

The colorimetric and fluorescence signaling properties of Res-DT for representative metal ions and anions were investigated under optimized conditions. First, the colorimetric signaling response of Res-DT was examined upon exposure to various common metal ions (Fig. 1). Res-DT exhibited a weak absorption band at 452 nm with pale-yellow coloration in the measurement solution. However, after treatment with Pd^2+^ ions, it showed a strong increase and moderate decrease in the absorbance at 572 and 452 nm, respectively. Concomitantly, the solution turned magenta from pale yellow (Fig. 1, inset). Furthermore, the other tested metal ions did not induce noticeable changes in the UV-vis spectra or color. Because the signaling ability of Res-DT for Pd^2+^ was related to the absorbance variation at two widely separated wavelengths, 572 nm and 452 nm, the selectivity for Pd^2+^ was assessed by ratiometry using the absorbance ratio (*A*_572_/*A*_452_) (Fig. 1). The *A*_572_/*A*_452_ value of Res-DT with Pd^2+^ was 10.2, whereas those of the other tested metal ions were remarkably low and similar to that of Res-DT alone (values ranging from 0.04 (Mg^2+^) to 0.11 (Pt^2+^)). Additionally, the Pd^2+^ selectivity of Res-DT was also confirmed in the presence of several representative anions (Fig. S4, ESI†) by showing the low *A*_572_/*A*_452_ values of Res-DT for anions (ranging from 0.06 (Cl^−^) to 0.08 (N_3_^−^)). Furthermore, taking into the fact that the dithioate-based probe has been used for hypochlorite sensing,^38^ the changes in the absorbance ratio of Res-DT upon treatment with representative oxidants were measured. The results (Fig. S5, ESI†) indicated that the tested oxidants, including hypochlorite ions, negligibly altered the *A*_572_/*A*_452_ ratio (ranging from 0.05 for perborate (PB) to 0.06 for *tert*-butyl hydroperoxide (TBHP)). The photophysical properties of Res-DT, both pre- and post-Pd^2+^ signaling, are detailed in Table S2 (ESI).†

**Fig. 2:**
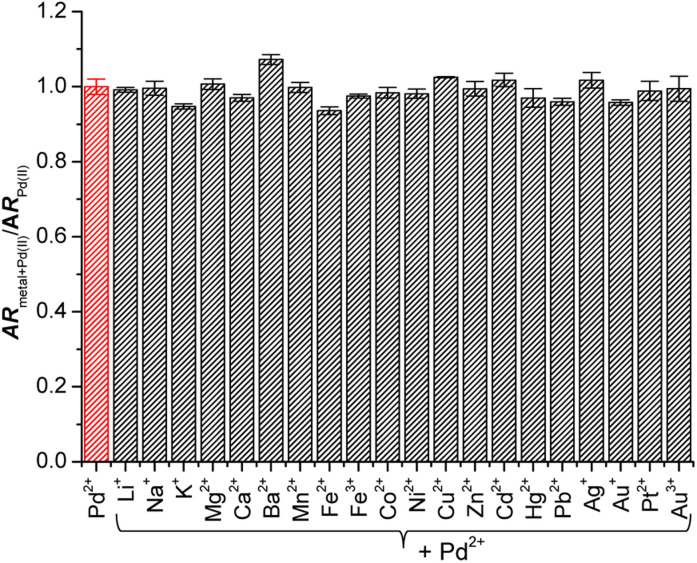
Effect of coexisting metal ions on the Pd^2+^ signaling activity of Res-DT. [Res-DT] = 5.0 μM, [Pd^2+^] = [M^*n*+^] = 25 μM, [SDS] = 10.0 mM, [PBS pH 7.4] = 10.0 mM in an aqueous solution containing 2% (v/v) DMSO. AR denotes the absorbance ratio *A*_572_/*A*_452_.

Next, to confirm the influence of background ions on the Pd^2+^ signaling tendency of Res-DT, sensing experiments with coexisting metal ions and anions were performed. According to the results (Fig. 2), the Pd^2+^ signaling behavior of Res-DT was unaffected by the presence of coexisting metal ions. Essentially, the *A*_572_/*A*_452_ value of the samples after the Pd^2+^ signaling experiments varied only slightly, ranging from 93.6% (for Fe^2+^) to 107.3% (for Ba^2+^) of the control result. In addition, the Pd^2+^ signaling performance of Res-DT was not altered by the presence of different anions, given the narrow range of the *A*_572_/*A*_452_ values (92.5% for N_3_^−^ to 103.0% for F^−^) (Fig. S6, ESI†).

**Fig. 3:**
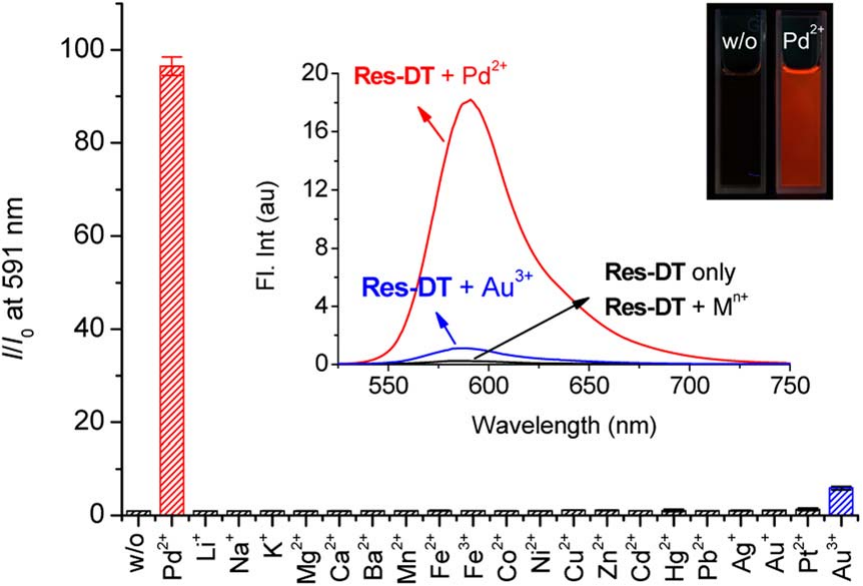
Changes in the fluorescence enhancement of Res-DT at 591 nm with the incorporation of common metal ions. Inset: fluorescence spectra and photographs with hand-held UV-lamp irradiation of Res-DT. [Res-DT] = 5.0 μM, [Pd^2+^] = [M^*n*+^] = 25 μM, [SDS] = 10.0 mM, [PBS pH 7.4] = 10.0 mM in an aqueous solution containing 2% (v/v) DMSO. *λ*_ex_ = 485 nm.

Resorufin-based sensing probes typically exhibit significant fluorescence features as well as remarkable colorimetric signaling behavior.^42^ Therefore, the Pd^2+^ signaling performance of Res-DT was evaluated based on the changes in the fluorescence response occurring under the same sensing conditions. Based on the results (Fig. 3, inset), Res-DT exhibited faint fluorescence emission at approximately 586 nm (*Φ*_Res-DT_ = 0.009) owing to the photophysical characteristics of the phenolic moiety-protected resorufin fluorophore.^43^ However, upon exposure to Pd^2+^ ions, Res-DT revealed strong fluorescence emission (*Φ*_Res-DT__+Pd(ii)_ = 0.58), exhibiting over a 90-fold fluorescence enhancement at 591 nm (Fig. 3). All other tested metal ions showed insignificant fluorescence responses except for Au^3+^ ions (*I*/*I*_0_ = 5.88), with *I*/*I*_0_ generally fluctuating between 0.97 (for Li^+^) and 1.32 (for Pt^2+^). Furthermore, no measurable changes were observed in the fluorescence emission of Res-DT toward the encountered anions, with *I*/*I*_0_ varying from 0.99 (for Cl^−^) to 1.50 (for N_3_^−^) (Fig. S7, ESI†). These results highlight the fluorometric potential of Res-DT to sense Pd^2+^ ions in chemical and industrial applications. However, the Pd^2+^ sensing behavior of Res-DT was investigated using colorimetric measurements which allowed the ratiometric analysis, rather than the method relying on a simple turn-on type fluorescence enhancement at a single wavelength. Ratiometry offers several advantages including increased sensitivity, improved selectivity, and reduced interference from the effects of interfering substances in the sample matrix.^44^

**Scheme 2:**
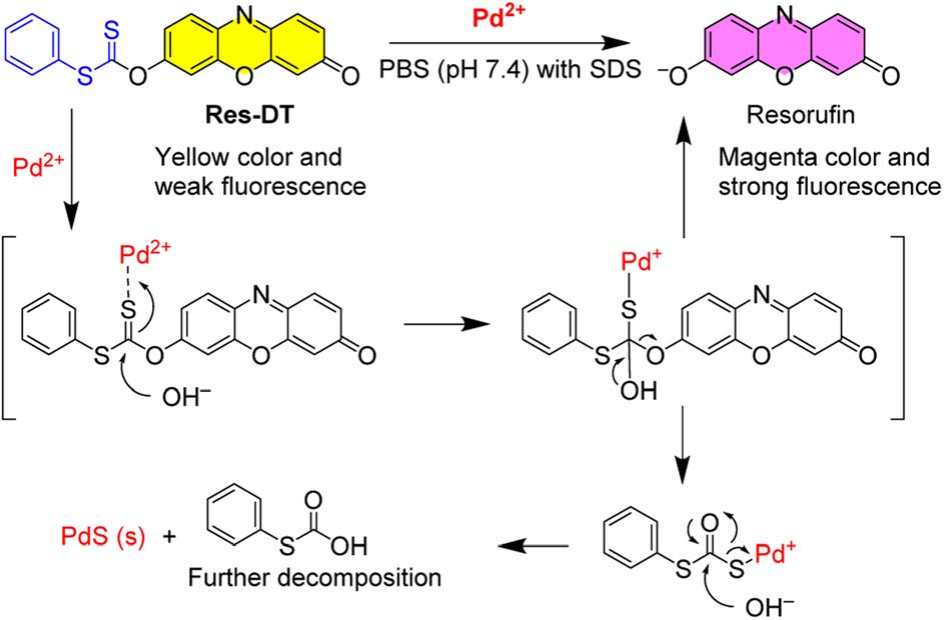
Suggested Pd^2+^ sensing mechanism of Res-DT.

**Fig. 4:**
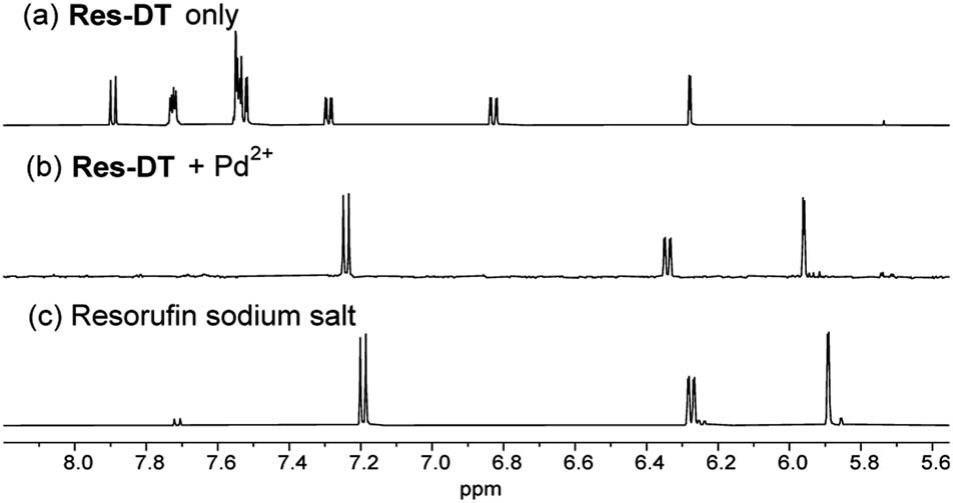
Partial ^1^H NMR spectra of (a) Res-DT, (b) purified Pd^2+^ signaling product of Res-DT (Res-DT + Pd^2+^), and (c) resorufin sodium salt in DMSO-*d*_6_. [Res-DT] = [resorufin sodium salt] = 10.0 mM. The spectrum of Res-DT + Pd^2+^ (b) was acquired after purifying the signaling product of Res-DT (10.0 mM) and Pd(OAc)_2_ (25.0 mM) in CH_3_CN.

The Pd^2+^ signaling was hypothesized to be caused by the generation of the resorufin fluorochrome *via* Pd^2+^-mediated hydrolysis of the dithioate moiety of Res-DT (Scheme 2). In the proposed sensing mechanism of Res-DT, the initial stage involves complex formation between the sulfur atom of the C=S bond and thiophilic Pd^2+^ ions.^45^ The complex is then hydrolyzed, yielding resorufin dye with its distinctive magenta color and strong fluorescence signals. To confirm the sensing mechanism of Res-DT, the Pd^2+^ signaling product was scrutinized using ^1^H NMR measurements. The results indicated that the NMR pattern of Res-DT was similar to that of typical phenol-protected resorufin compounds (Fig. 4).^40^ Moreover, the purified Pd^2+^ sensing product (Res-DT + Pd^2+^) exhibited three well-defined resonances in the ^1^H NMR spectrum that were likely associated with resorufin. Furthermore, our investigation using thin-layer chromatography of the Pd^2+^ signaling solution of Res-DT revealed that resorufin is produced (Fig. S8, ESI†). Through FAB mass spectrometry, a diagnostic peak at *m*/*z* = 214 was identified in the Pd^2+^ signaling product, which matches the calculated mass of resorufin (C_12_H_8_NO_3_^+^ [M + H] = 214) (Fig. S9, ESI†). Additionally, we isolated a greenish-black colored precipitate from the Pd^2+^ signaling solution and confirmed it to be PdS using FE-SEM equipped with EDX (Fig. S10, ESI†).

**Fig. 5:**
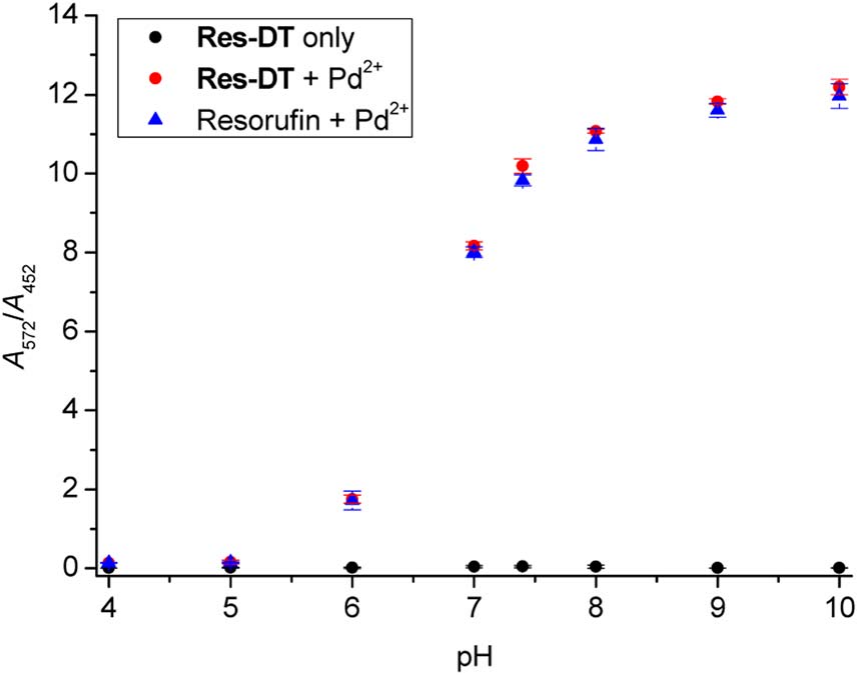
Effect of pH on the Pd^2+^ signaling of Res-DT, represented by the absorbance ratio *A*_572_/*A*_452_. [Res-DT] = [resorufin] = 5.0 μM, [Pd^2+^] = 25 μM, [SDS] = 10.0 mM, [PBS pH 7.4] = 10.0 mM in an aqueous solution containing 2% (v/v) DMSO. The pH was adjusted using 0.1 N HCl and NaOH.

The influence of pH on the Pd^2+^ signaling performance of Res-DT was tested to assess its practical usability. The results showed that the *A*_572_/*A*_452_ value of pristine Res-DT remained constant across the pH range 4.0–10.0 (Fig. 5). In contrast, that of the Pd^2+^ signaling sample (Res-DT + Pd^2+^) increased significantly from pH 6.0 onward and stabilized at around pH 8; this tendency was mirrored by that of the reference compound resorufin in the presence of coexisting Pd^2+^ ions. This finding suggested that the pH profile of the Pd^2+^ signaling was due to the pH dependency of the spectroscopic properties of resorufin. Next, to verify the signaling performance of Res-DT for Pd species with different oxidation states, the response of the probe to Pd(ii)OAc_2_, Pd(ii)Cl_2_, Pd(0)(PPh_3_)_4_, and K_2_Pd(iv)Cl_6_ was monitored. The results indicated that Res-DT exhibited similar signaling behavior, as evidenced by the absorbance ratios, for the different Pd species in the employed pH 7.4 PBS solution (Fig. S11, ESI†).

**Fig. 6:**
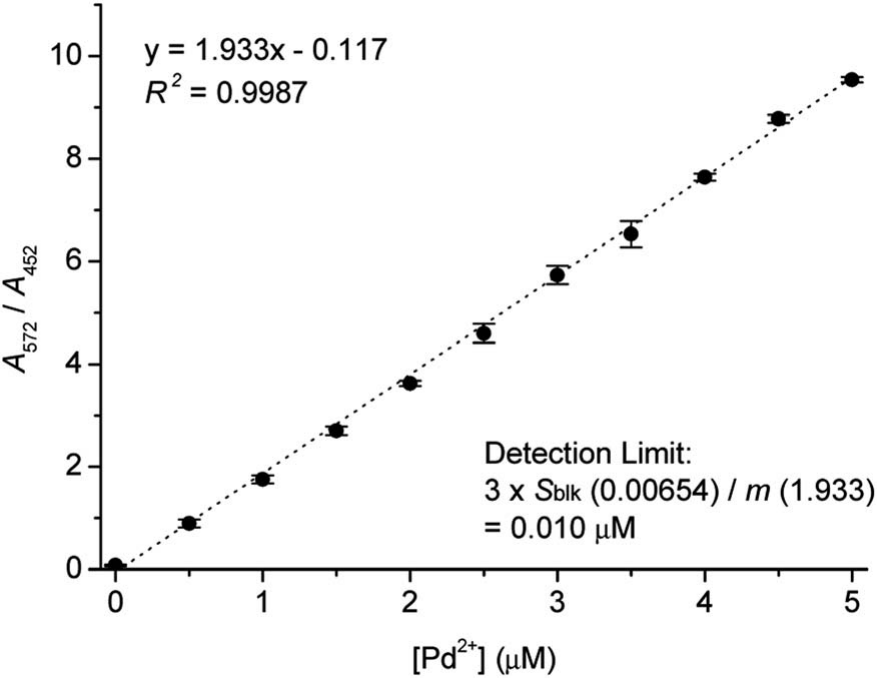
Calibration curve for Pd^2+^ determination using the absorbance ratio *A*_572_/*A*_452_. [Res-DT] = 5.0 μM, [Pd^2+^] = 0–5.0 μM, [SDS] = 10.0 mM, [PBS pH 7.4] = 10.0 mM in an aqueous solution containing 2% (v/v) DMSO.

UV-vis titration of Res-DT with Pd^2+^ was subsequently performed to determine the minimum Pd^2+^ ion concentration detected using Res-DT. The absorbance ratio *A*_572_/*A*_452_ increased linearly up to a Pd^2+^ concentration of 5.0 μM (*R*^2^ = 0.9987) (Fig. 6). Using the titration plot and IUPAC recommended equation (3*s*_blk_/*m*), where *s*_blk_ and *m* denote standard deviation of the blank signal and analytical sensitivity, respectively, the detection limit was calculated to be 10 nM.^46^ Additionally, we studied the quantitative analytical behavior of Res-DT for Pd^2+^ sensing by fluorescence titration (Fig. S12, ESI†). The fluorescence emission changes at 591 nm demonstrated a linear correlation with increasing concentrations of Pd^2+^ (*R*^2^ = 0.9870). From these results, the detection limit for Pd^2+^ ions was calculated to be 7.3 nM.^46^ Furthermore, we estimated the detection time of Res-DT for Pd^2+^ ions by observing the time-dependent change in the absorbance ratio (*A*_572_/*A*_452_), and it was found to be 7 min (Fig. S13, ESI†).

**Fig. 7:**
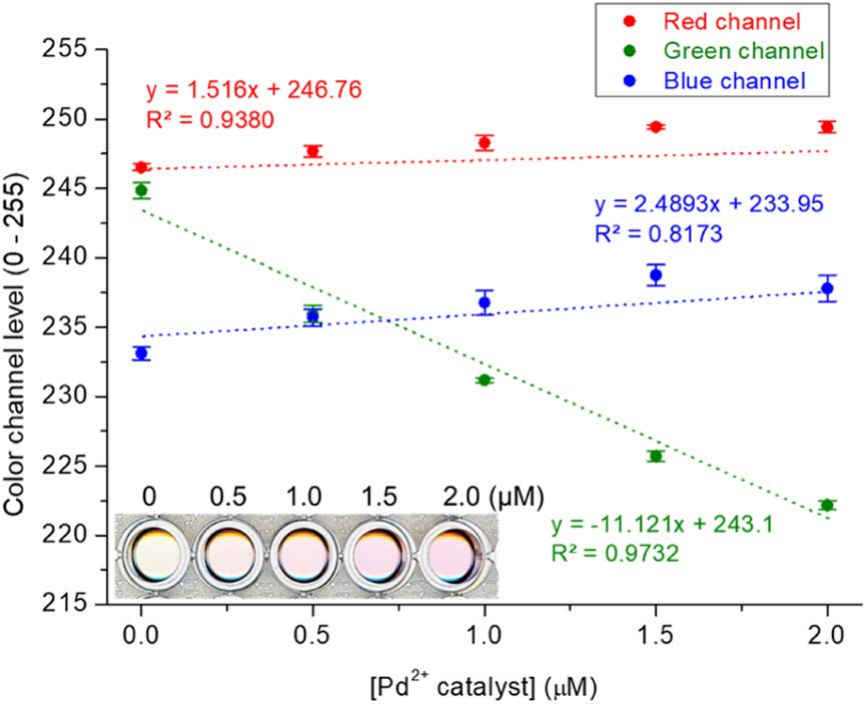
Plot illustrating the color channel level changes (RGB) in response to a Pd-containing catalyst. Inset: images of solutions with different Pd^2+^ concentrations captured using a scanner. [Res-DT] = 5.0 μM, [Pd^2+^ catalyst] = 0–2.0 μM, [SDS] = 10.0 mM, [PBS pH 7.4] = 10.0 mM in an aqueous solution containing 2% (v/v) DMSO.

Finally, to assess the practical applicability of the devised probe, Pd assay in Pd-containing catalyst and drug candidate was performed using an office scanner as a readily accessible device for detection.^47^ We used the white catalyst as the Pd-containing catalyst, and a Pd^2+^–2-picolinic acid complex as the Pd-containing drug candidate.^48^ The Res-DT with the tested catalyst and drug candidate exhibited a noticeable color shift from yellow to magenta, and the variation in color could be conveniently characterized through analysis of the RGB color channel levels of the scanned image (Fig. 7 and S14, ESI†). Consequently, acceptable calibration curves based on the green channel, rather than the red and blue channels, were obtained for the catalyst and drug candidate, which yielded satisfactory *R*^2^ values of 0.9732 and 0.9887 for the catalyst and drug candidate, respectively. The results of the colorimetric signaling-based Pd^2+^ assay for the catalyst and drug candidate performed using the office scanner (recovery = 94.1–107.2%) were consistent with those of UV-vis spectrometry (recovery = 91.0–101.3%) (Table S3, ESI†). This result implies that the Res-DT could be successfully applied to the analysis of Pd^2+^ ions in palladium relevant catalysts and drugs.

## 4. Conclusions

A simple colorimetric and fluorescent probe (Res-DT) was developed for the convenient determination of Pd species in Pd-containing catalyst and drug candidate. Res-DT exhibited selective, sensitive signaling behavior toward Pd species without interference from common metal ions, anions, and oxidants. The signaling was achieved by Pd-induced hydrolysis of the dithioate moiety of Res-DT, generating resorufin dye that exhibits a magenta color, visible to the naked eye, and intense fluorescence. The probe was immune to interference from several other metal ions and anions when detecting Pd ions. Additionally, the Pd signaling was achieved within 7 min, and the detection limit of the probe was determined to be 10 nM. Finally, Res-DT was successfully employed to analyze Pd^2+^ ions in Pd-containing catalyst and drug candidate. The designed Res-DT can potentially be applied in various practical and industrial settings featuring Pd-related systems.

## Conflicts of interest

There are no conflicts of interest to declare.

## Supplementary Material

RA-013-D3RA05549C-s001
