# Supplementary material for: A colorimetric and fluorescent signaling probe for assaying Pd2+ in practical samples
Source: RSC Adv. 2023 Nov 1;13(45):31962–8. doi: 10.1039/d3ra05549c (PMC10618942; doi:10.1039/d3ra05549c)
Supplement: RA-013-D3RA05549C-s001 [file RA-013-D3RA05549C-s001.pdf]

Electronic Supplementary Material (ESI) for RSC Advances.
This journal is © The Royal Society of Chemistry 2023

Electronic Supplementary Information

A colorimetric and fluorescent signaling probe for assaying Pd^{2+} in practical samples

Myung Gil Choi, Juyoung Han, Sangdoo Ahn* and Suk-Kyu Chang*

Department of Chemistry, Chung-Ang University, Seoul 06974, Republic of Korea

Contents

Experimental details

Table S1. Representative Pd-selective reaction-based probes reported previously
Table S2. Photophysical properties of **Res-DT** in the presence and absence of Pd^{2+} ions
Table S3. Analysis of Pd^{2+} ions in a Pd-containing catalyst and a drug candidate

Fig. S1. Time-dependent Pd^{2+} signaling performance of **Res-DT**.
Fig. S2. Time-dependent Pd^{2+} signaling behavior of **Res-DT** in solutions containing (a) SDS, (b) CTAB, or (c) Tween 20 as a signal-boosting surfactant.
Fig. S3. Precipitation profile illustrating the Pd^{2+} signaling of **Res-DT** in solutions containing SDS or Tween 20 as the surfactant under illumination with a red laser.
Fig. S4. Changes in the absorbance ratio of **Res-DT** (A_{572}/A_{452}) in the presence of common anions.
Fig. S5. Changes in the absorbance ratio of **Res-DT** (A_{572}/A_{452}) in the presence of common oxidants.
Fig. S6. Effect of competitive anions on the Pd^{2+} signaling performance of **Res-DT**.
Fig. S7. Changes in the fluorescence enhancement of **Res-DT** at 591 nm with the incorporation of common anions. Inset: fluorescence spectra of **Res-DT**.
Fig. S8. TLC profile of **Res-DT** (spot A), **Res-DT** with Pd^{2+} (spot B), and resorufin

(spot C).
Fig. S9. FAB mass spectrum of the Pd^{2+} signaling product of **Res-DT**.
Fig. S10. FE-SEM image and associated EDX maps and data of the precipitate formed from the Pd^{2+} signaling of **Res-DT**.
Fig. S11. Changes in the absorbance ratio of **Res-DT** (A_{572}/A_{452}) in the presence of diverse Pd species.
Fig. S12. Calibration curve for Pd^{2+} determination using the fluorescence emission at 591 nm.
Fig. S13. Time-dependent Pd^{2+} signaling activity of **Res-DT**, as characterized by the absorbance ratio of the signaling solution (A_{572}/A_{452}).
Fig. S14. Plot illustrating the changes in color channel levels (RGB) in the presence of a Pd-containing drug candidate.
Fig. S15. ^{1}H NMR spectrum of **Res-DT** in $CDCl_3$ (600 MHz).
Fig. S16. ^{13}C NMR spectrum of **Res-DT** in $CDCl_3$ (150 MHz).
Fig. S17. High-resolution mass spectrum of **Res-DT**.

Experimental details

1. General

Various materials including resorufin, phenyl chlorodithioformate, and Pd species such as $Pd(OAc)_2$, $PdCl_2$, $Pd(PPh_3)_4$, and K_2PdCl_6 were obtained from Merck KGaA. The White catalyst (1,2-bis(phenylsulfinyl)ethane palladium(II) acetate) and 2-picolinic acid were also sourced from Merck KGaA and were used without further purification. ^{1}H and ^{13}C NMR measurements were performed using a Varian VNS NMR spectrometer. High-resolution mass spectrometry was conducted using a JEOL JMS-700 mass spectrometer with Fast atom bombardment (FAB) ionization. UV–vis and fluorescence signaling behavior were examined using Scinco S-3100 and FS-2 spectrophotometers, respectively. FE-SEM and EDX map images were obtained using Carl Zeiss SIGMA 300 microscopy equipped with Oxford EDX spectroscopy. The Pd-containing drug candidate was prepared according to a previously reported method.[S1]

Table S1. Representative Pd-selective reaction-based probes reported previously

Mechanism	Structure	Signal	Conditions	LOD	Application	Merit	Ref.
Deallylation	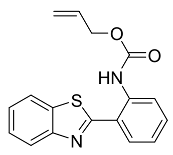	Fluorescence	CH_3CN:PBS buffer (8:2, *v/v*, pH 7.4)	2.6 nM	Pd^{2+} assay in tap water, river water, and wastewater	● Environmental Pd sensing<br>● Low LOD	S2
	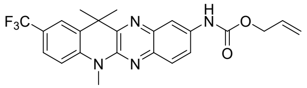	Colorimetry, fluorescence	PEG400:PBS buffer (6:4, *v/v*, pH 7.4)	29.4 nM	Pd imaging in HeLa cells	● Naked-eye detectable<br>● Bioimaging suitable	S3
	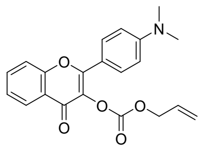	Fluorescence	THF:PBS buffer (8:2, *v/v*, pH 7.4)	9.0 nM	Pd imaging in HeLa cells	● Bioimaging suitable<br>● Low LOD	S4
	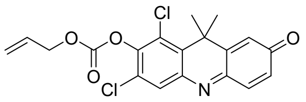	Colorimetry, fluorescence	DMSO:PBS buffer (8:2, *v/v*, pH 7.4)	2.2 nM	Pd^{2+} imaging in HeLa cells	● Naked-eye detectable<br>● Bioimaging suitable<br>● Low LOD	S5
	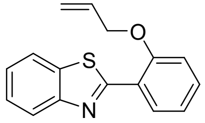	Fluorescence	CH_3CN:HEPES buffer (8:2, *v/v*, pH 7.2)	40 nM	Pd^{2+} imaging in PC3 cells	● Bioimaging suitable	S6
	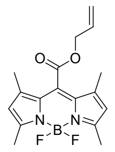	Colorimetry, fluorescence	pH 7.4 phosphate buffer with 1% DMSO	7.4 nM	Pd imaging in HeLa cells	● Naked-eye detectable<br>● Bioimaging suitable<br>● Low LOD	S7

Table S1. Representative Pd-selective reaction-based probes reported previously (*continued*)

Mechanism	Structure	Signal	Conditions	LOD	Application	Merit	Ref.
Depropargylation	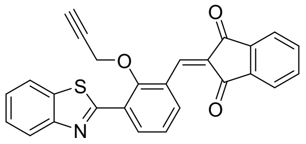	Colorimetry, fluorescence	THF:HEPES buffer (5:2, *v/v*, pH 7.4)	14.6 nM	Fabricating a paper-based test strip	● Naked-eye detectable<br>● Paper strip	S8
	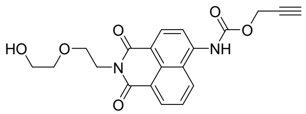	Fluorescence	pH 7.4 PBS buffer with 0.5% EtOH	25 nM	Pd^{2+} imaging in Hep G2 and HL60 cells	● Bioimaging suitable	S9
Claisen rearrangement	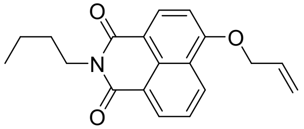	Fluorescence	pH 10.0 Na_2CO_3/$NaHCO_3$ buffer	1.4 μM	Pd^{2+} sensing in a three-way catalyst	● Industrial Pd sensing<br>● Low LOD	S10
Dimerization	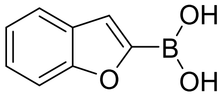	Fluorescence	CH_3CN under basic conditions	9.8 nM	Pd^{2+} assay in tap water and river water	● Environmental Pd sensing<br>● Low LOD	S11
Oxidative cyclization	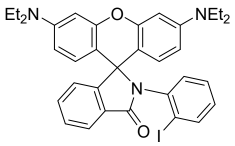	Colorimetry, fluorescence	CH_3CN	940 nM	Residual Pd detection in reactors	● Naked-eye detectable<br>● Industrial Pd sensing	S12
Hydrolysis	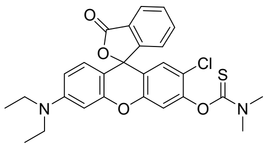	Colorimetry, fluorescence	DMSO:phosphate buffer (1:1, *v/v*, pH 7.0)	10 nM	Pd^{2+} assay in a real drug (ibuprofen) and a drug intermediate	● Naked-eye detectable<br>● Pharmaceutical analysis<br>● Low LOD	S13

Table S1. Representative Pd-selective reaction-based probes reported previously (*continued*)

Mechanism	Structure	Signal	Conditions	LOD	Application	Merit	Ref.
Hydrolysis	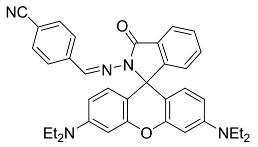	Colorimetry, fluorescence	CH_3CN:H_2O (3:1, *v/v*)	57 nM	Pd^{2+} imaging in MCF7 cells	● Naked-eye detectable<br>● Bioimaging suitable	S14
	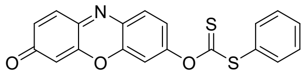	Colorimetry, fluorescence	pH 7.4 PBS buffer with 2% DMSO	10 nM	Pd^{2+} assay in a Pd-containing catalyst and a drug candidate	● Naked-eye detectable<br>● Pharmaceutical analysis<br>● Industrial Pd sensing<br>● Scanner-assisted sensing<br>● Low LOD	This work

Table S2. Photophysical properties of **Res-DT** in the presence and absence of Pd^{2+} ions

Sample	Absorbance		Fluorescence	
	λ_{max}	Molar extinction coefficient (ε)	λ_{max}	Quantum yield (Φ)
**Res-DT**	452 nm	12,300 $M^{-1}\cdot cm^{-1}$	586 nm	0.009
**Res-DT** + Pd^{2+}	572 nm	64,500 $M^{-1}\cdot cm^{-}$	591 nm	0.58

Table S3. Analysis of Pd^{2+} ions in a Pd-containing catalyst and a drug candidate

Analyte	Pd^{2+} added (μM)	Method involving UV–vis spectrometry		Method featuring an office scanner	
		Pd^{2+} detected (μM, *n* = 3)	Recovery	Pd^{2+} detected (μM, *n* = 3)	Recovery
Pd-containing catalyst (the White catalyst)	0	Not detected	-	Not detected	-
	1.0	0.92 ± 0.02	92.0%	1.07 ± 0.01	107.2%
	2.0	2.02 ± 0.04	101.3%	1.88 ± 0.03	94.1%
Pd-containing drug candidate	0	Not detected	-	Not detected	-
	1.0	0.91 ± 0.02	91.0%	1.04 ± 0.01	103.8%
	2.0	1.99 ± 0.05	99.8%	1.94 ± 0.02	97.4%

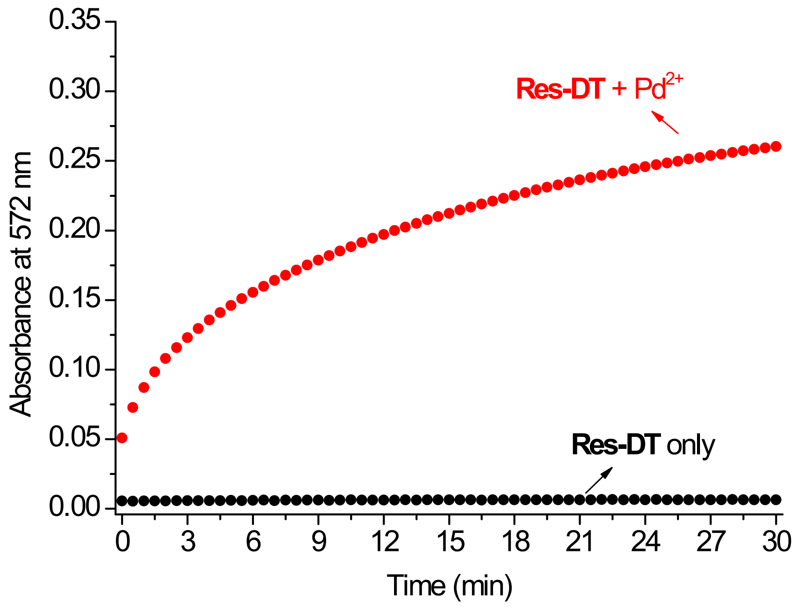

Fig. S1. Time-dependent Pd^{2+} signaling performance of **Res-DT**. [**Res-DT**] = 5.0 μM, [Pd^{2+}] = 25 μM, [PBS pH 7.4] = 10.0 mM in an aqueous solution containing 2% (*v/v*) DMSO.

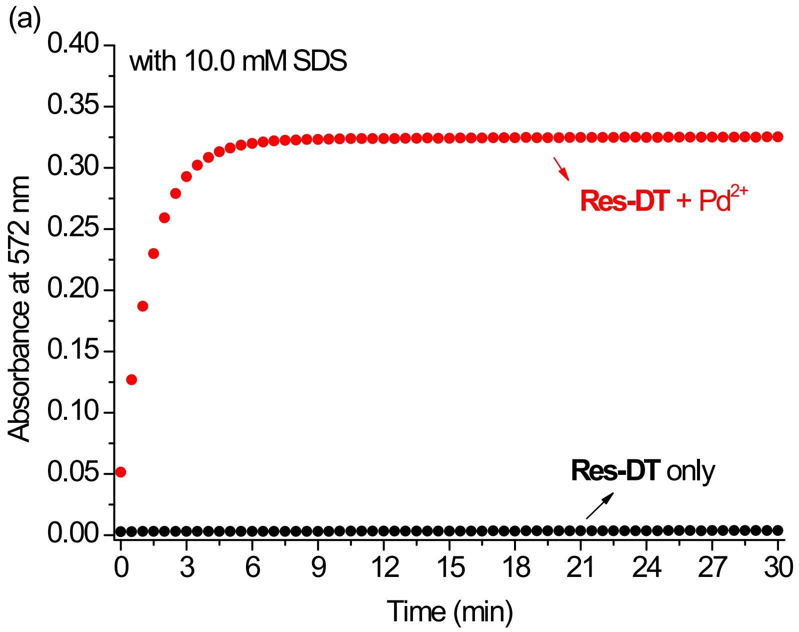

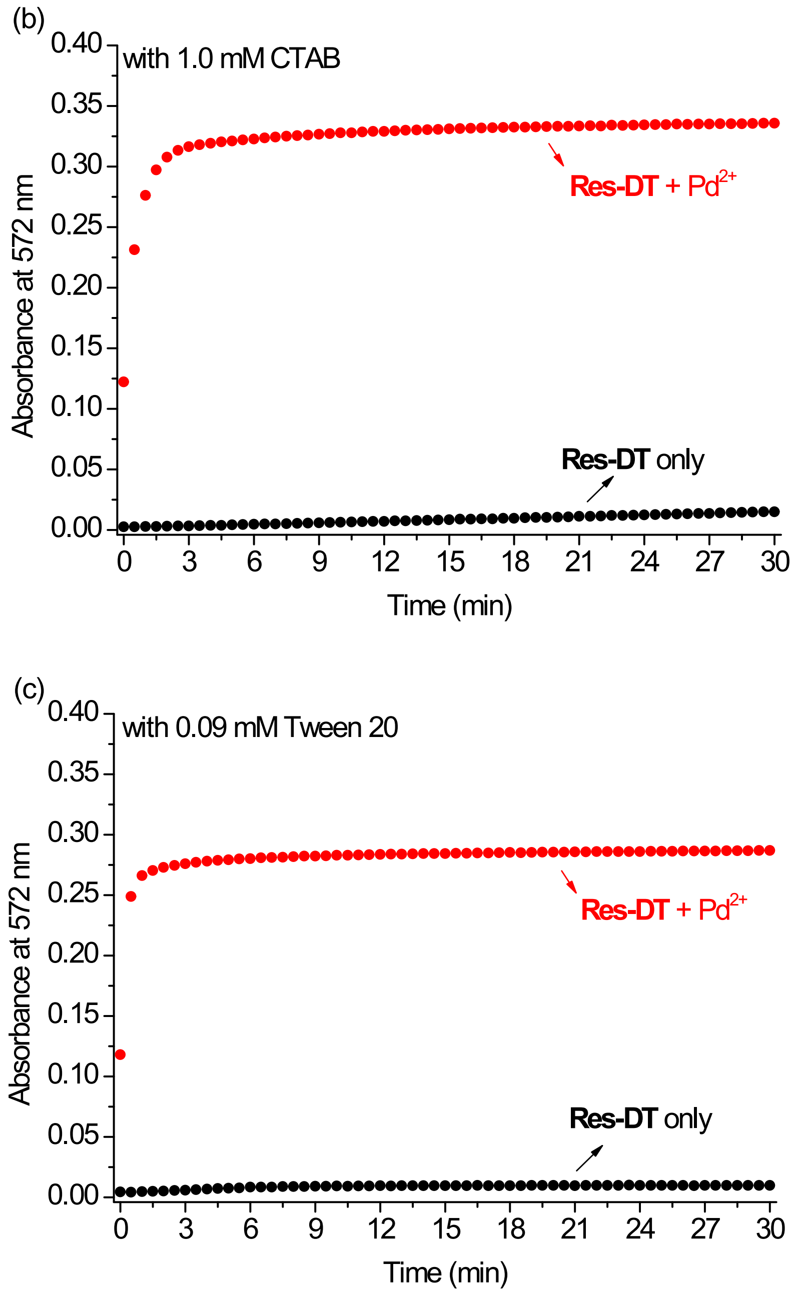

Fig. S2. Time-dependent Pd^{2+} signaling behavior of **Res-DT** in solutions containing (a) SDS, (b) CTAB, or (c) Tween 20 as a signal-boosting surfactant. [**Res-DT**] = 5.0 μM, [Pd^{2+}] = 25 μM, [SDS] = 10.0 mM, [CTAB] = 1.0 mM, [Tween 20] = 0.09 mM, [PBS pH 7.4] = 10.0 mM in an aqueous solution containing 2% (*v/v*) DMSO.

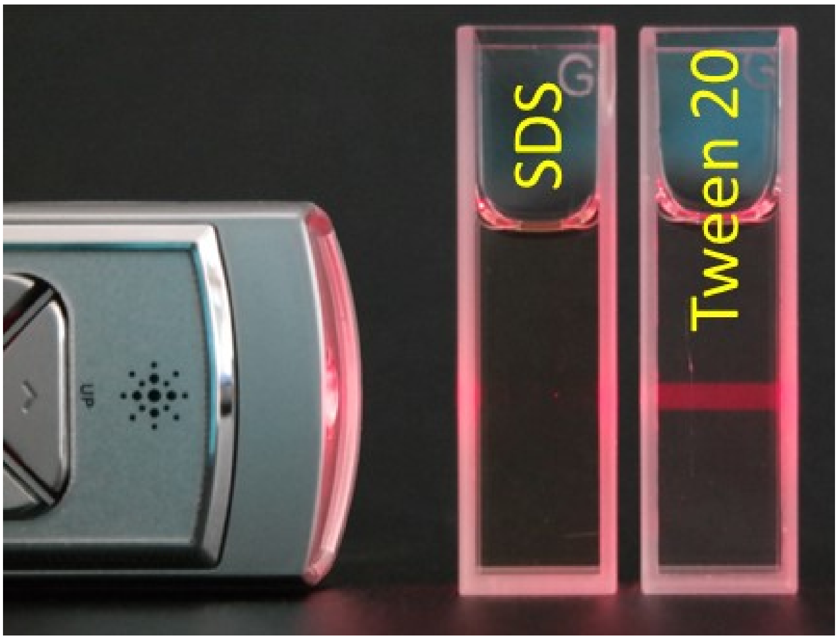

Fig. S3. Precipitation profile illustrating the Pd^{2+} signaling of **Res-DT** in solutions containing SDS or Tween 20 as the surfactant under illumination with a red laser. [**Res-DT**] = 5.0 μM, [Pd^{2+}] = 25 μM, [SDS] = 10.0 mM, [Tween 20] = 0.09 mM, [PBS pH 7.4] = 10.0 mM in an aqueous solution containing 2% (*v/v*) DMSO.

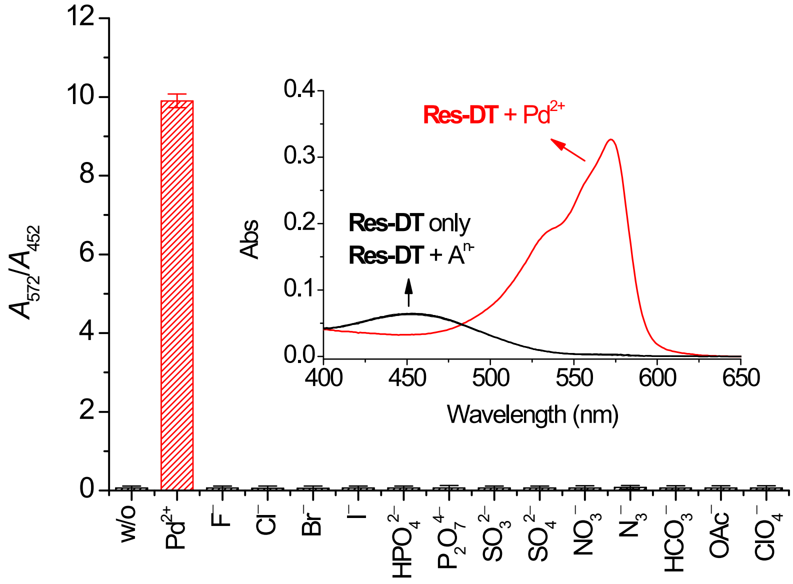

Fig. S4. Changes in the absorbance ratio of **Res-DT** (A_{572}/A_{452}) in the presence of common anions. Inset: UV–vis spectra of **Res-DT**. [**Res-DT**] = 5.0 μM, [Pd^{2+}] = [A^{n-}] = 25 μM, [SDS] = 10.0 mM, [PBS pH 7.4] = 10.0 mM in an aqueous solution containing 2% (*v/v*) DMSO.

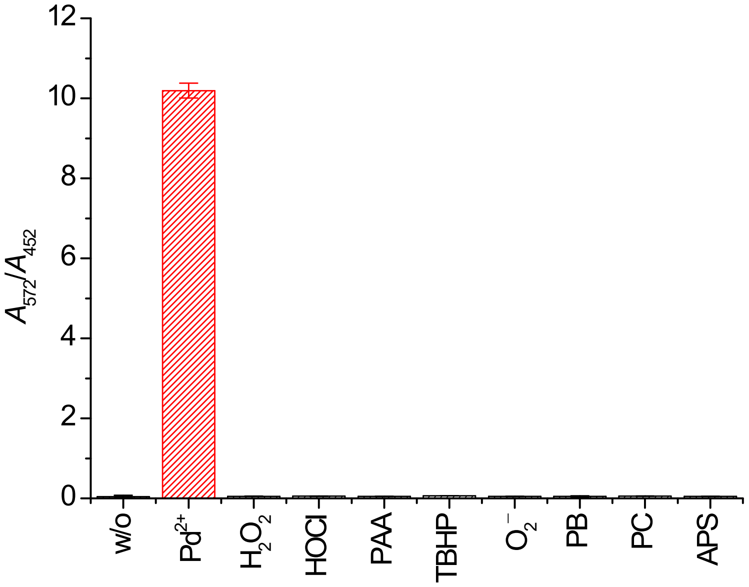

Fig. S5. Changes in the absorbance ratio of **Res-DT** (A_{572}/A_{452}) in the presence of common oxidants. [**Res-DT**] = 5.0 μM, [Pd^{2+}] = [oxidant] = 25 μM, [SDS] = 10.0 mM, [PBS pH 7.4] = 10.0 mM in an aqueous solution containing 2% (*v/v*) DMSO. PAA = peracetic acid, TBHP = *tert*-butyl hydroperoxide, PB = sodium perborate, PC = sodium percarbonate, APS = ammonium persulfate.

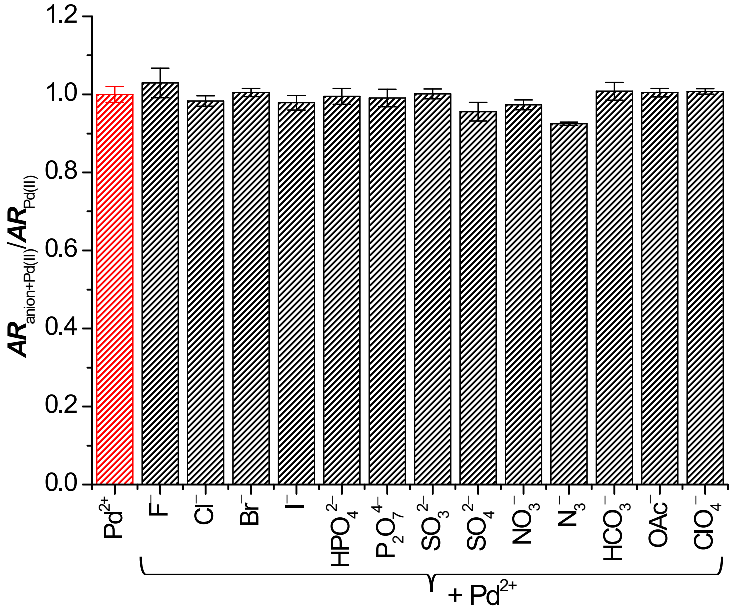

Fig. S6. Effect of competitive anions on the Pd^{2+} signaling performance of **Res-DT**. [**Res-DT**] = 5.0 μM, [Pd^{2+}] = [A^{n-}] = 25 μM, [SDS] = 10.0 mM, [PBS pH 7.4] = 10.0 mM in an aqueous solution containing 2% (*v/v*) DMSO. **AR** denotes the absorbance ratio A_{572}/A_{452}.

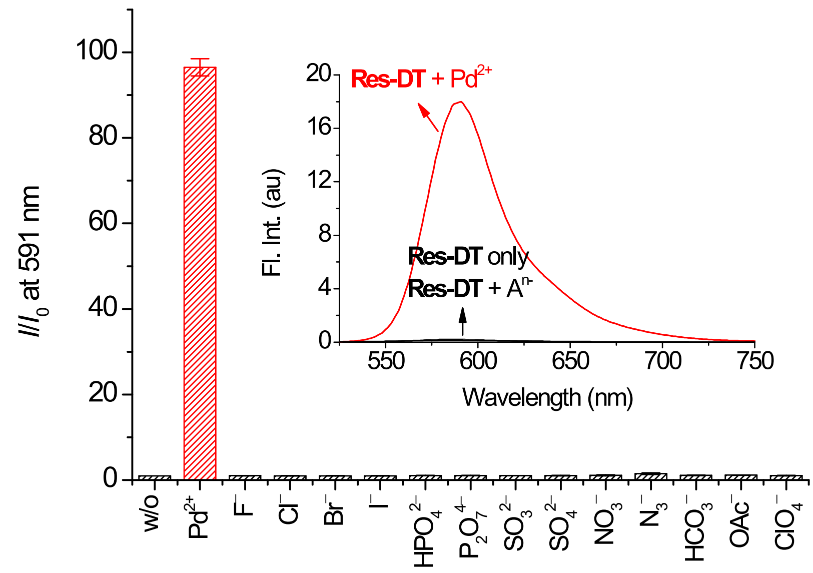

Fig. S7. Changes in the fluorescence enhancement of **Res-DT** at 591 nm with the incorporation of common anions. Inset: fluorescence spectra of **Res-DT**. [**Res-DT**] = 5.0 μM, [Pd^{2+}] = [A^{n-}] = 25 μM, [SDS] = 10.0 mM, [PBS pH 7.4] = 10.0 mM in an aqueous solution containing 2% (*v*/*v*) DMSO. λ_{ex} = 485 nm.

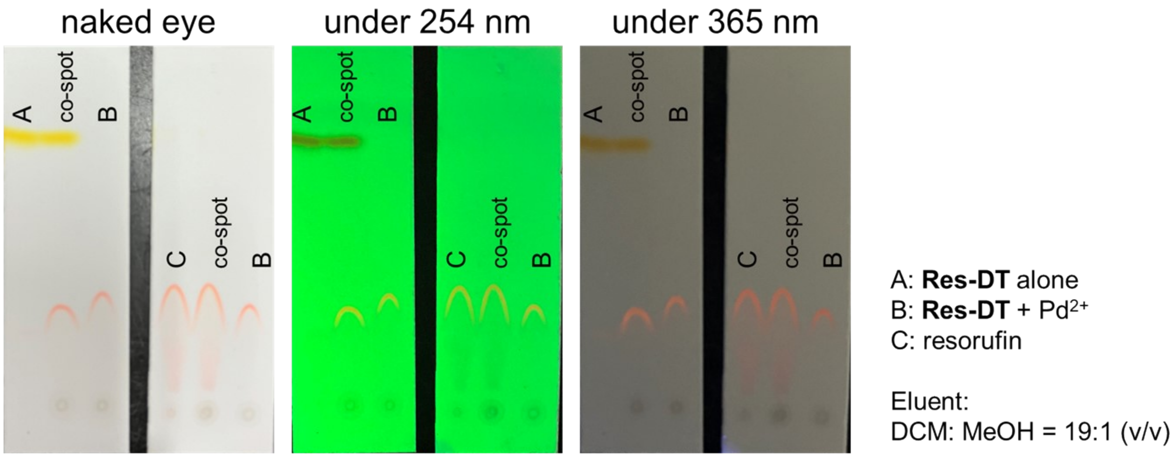

Fig. S8. TLC profile of **Res-DT** (spot A), **Res-DT** with Pd^{2+} (spot B), and resorufin (spot C).

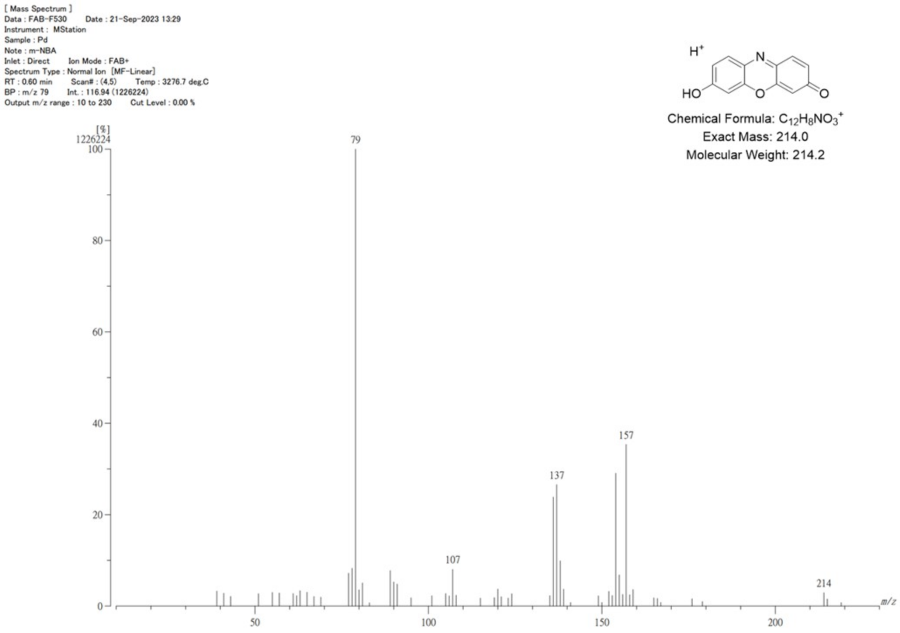

Fig. S9. FAB mass spectrum of the Pd^{2+} signaling product of **Res-DT**.

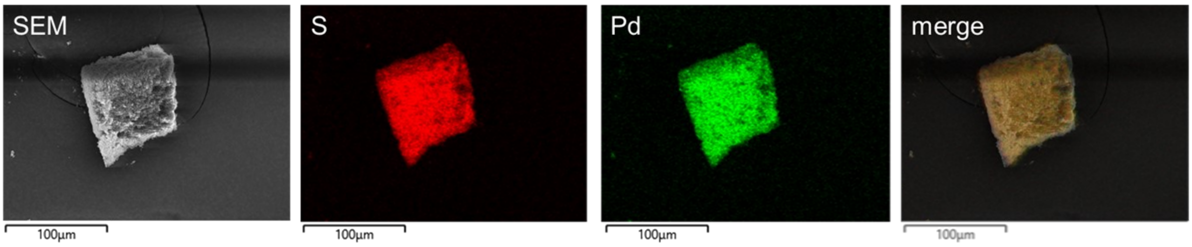

Element	Line Type	Apparent Concentration	k Ratio	Wt%	Wt% Sigma	Atomic%	Standard Label	Factory Standard
S	K series	24.02	0.20688	27.44	0.29	55.6	FeS2	Yes
Pd	L series	44.14	0.44143	72.56	0.29	44.4	Pd	Yes
Total:				100.00				

Fig. S10. FE-SEM image and associated EDX maps and data of the precipitate formed from the Pd^{2+} signaling of **Res-DT**.

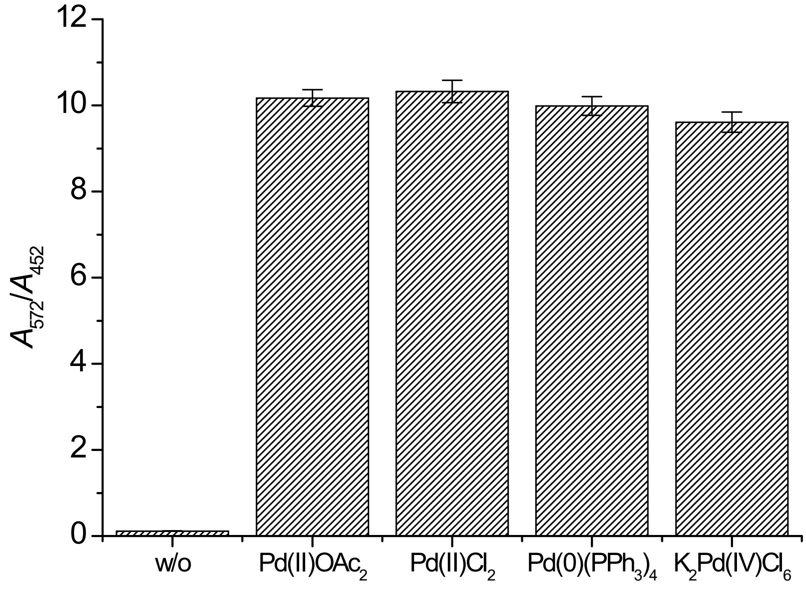

Fig. S11. Changes in the absorbance ratio of **Res-DT** (A_{572}/A_{452}) in the presence of diverse Pd species. [**Res-DT**] = 5.0 μM, [Pd species] = 25 μM, [SDS] = 10.0 mM, [PBS pH 7.4] = 10.0 mM in an aqueous solution containing 2% (*v/v*) DMSO.

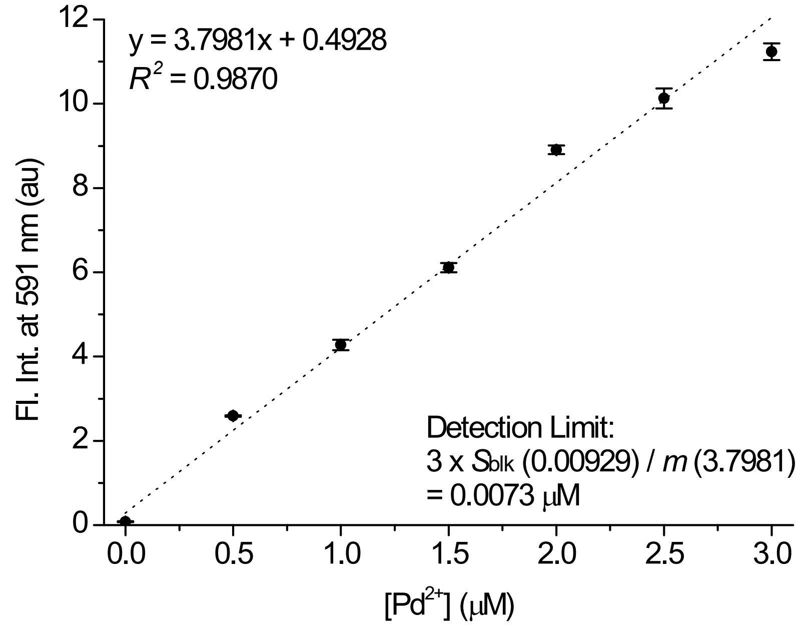

Fig. S12. Calibration curve for Pd^{2+} determination using the fluorescence emission at 591 nm. [**Res-DT**] = 5.0 μM, [Pd^{2+}] = 0–3.0 μM, [SDS] = 10.0 mM, [PBS pH 7.4] = 10.0 mM in an aqueous solution containing 2% (*v/v*) DMSO. λ_{ex} = 485 nm.

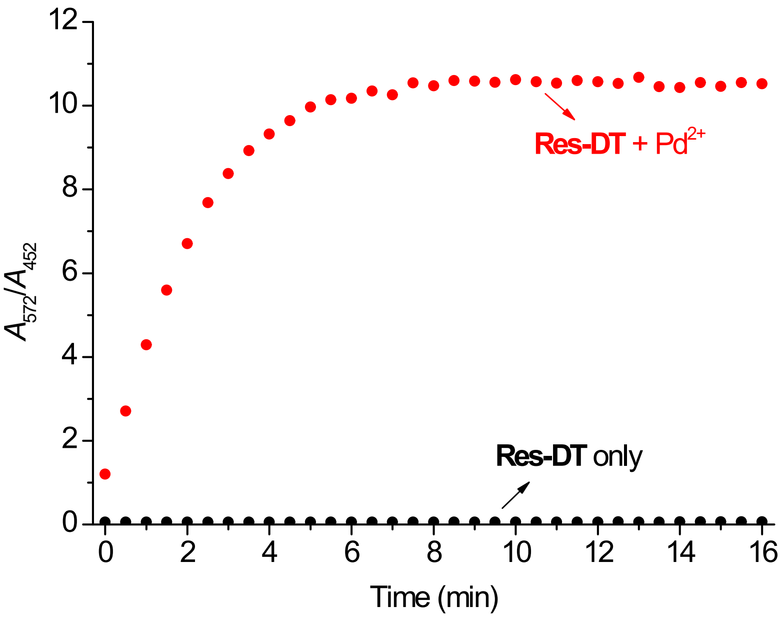

Fig. S13. Time-dependent Pd^{2+} signaling activity of **Res-DT**, as characterized by the absorbance ratio of the signaling solution (A_{572}/A_{452}). [**Res-DT**] = 5.0 μM, [Pd^{2+}] = 25 μM, [SDS] = 10.0 mM, [PBS pH 7.4] = 10.0 mM in an aqueous solution containing 2% (*v/v*) DMSO.

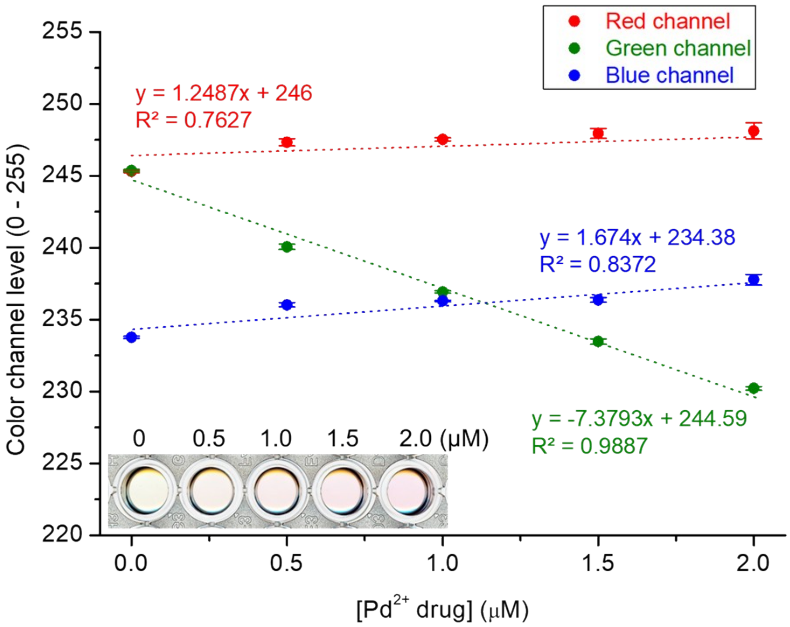

Fig. S14. Plot illustrating the changes in color channel levels (RGB) in the presence of a Pd-containing drug candidate. Inset: images of solutions with different Pd^{2+} concentrations

captured using a scanner. [**Res-DT**] = 5.0 μM, [Pd^{2+} drug] = 0–2.0 μM, [SDS] = 10.0 mM, [PBS pH 7.4] = 10.0 mM in an aqueous solution containing 2% (*v/v*) DMSO.

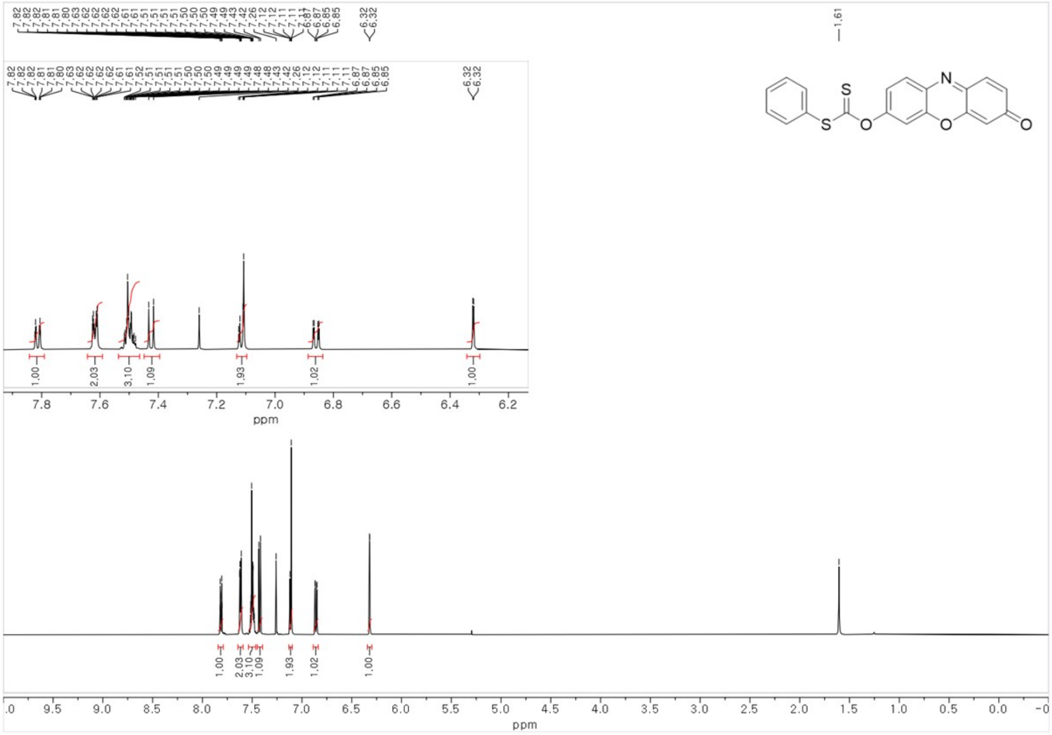

Fig. S15. ^{1}H NMR spectrum of **Res-DT** in $CDCl_3$ (600 MHz).

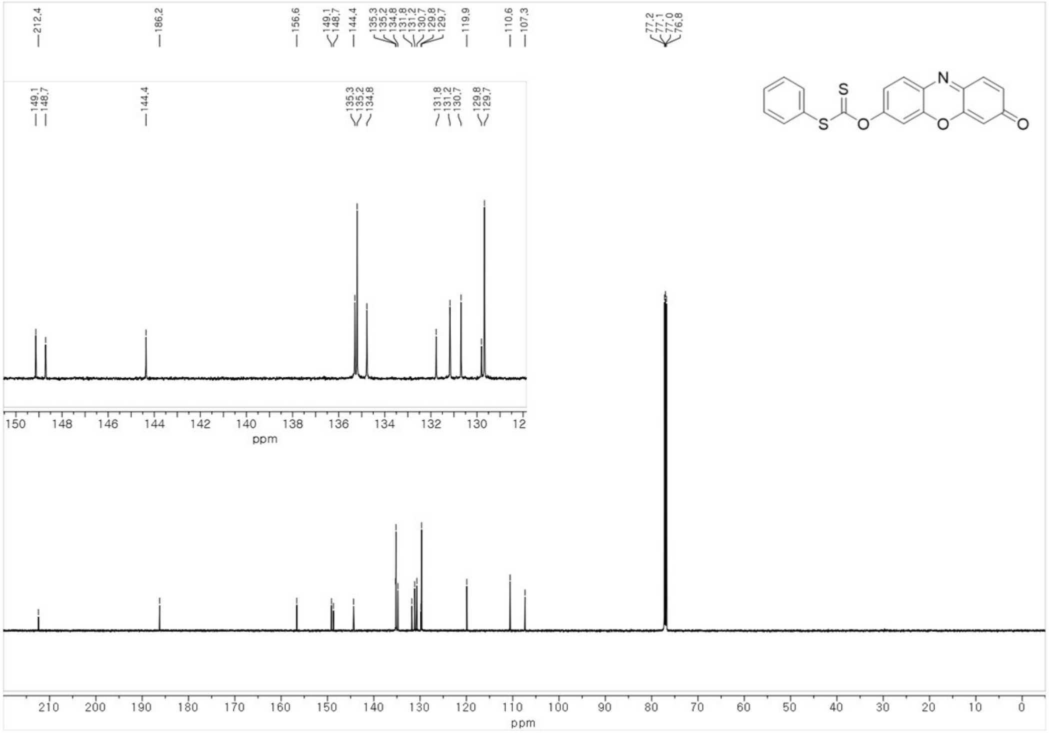

Fig. S16. ^{13}C NMR spectrum of **Res-DT** in $CDCl_3$ (150 MHz).

[Mass Spectrum]
Data : EI-C338 Date : 05-Oct-2022 17:44
RT : 0.79 min Scan# : (24,33)
Elements : C 100/0, H 100/0, N 3/0, O 10/0, S 3/0
Mass Tolerance : 10ppm, 5mmu if m/z < 500, 10mmu if m/z > 1000
Unsaturation (U.S.) : 10.0 - 25.0

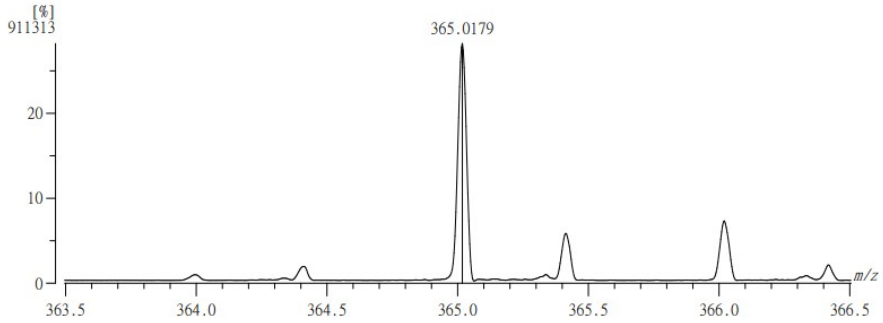

	Observed m/z	Int%	Err[ppm / mmu]	U.S.	Composition
1	365.0179	28.22	-12.7 / -4.6	25.0	C24 H3 N3 O2
2			-5.3 / -1.9	20.5	C21 H5 N2 O5
3			+2.0 / +0.7	16.0	C18 H7 N O8
4			+13.0 / +4.8	12.0	C13 H7 N3 O10
5			+8.9 / +3.2	21.0	C22 H7 N O3 S
6			-7.2 / -2.6	12.0	C15 H11 N O8 S
7			-7.7 / -2.8	21.5	C22 H9 N2 S2
8			-0.4 / -0.1	17.0	C19 H11 N O3 S2
9			+10.6 / +3.9	13.0	C14 H11 N3 O5 S2
10			+7.0 / +2.5	12.5	C16 H13 O6 S2
11			-9.6 / -3.5	13.0	C16 H15 N O3 S3

Fig. S17. High-resolution mass spectrum of **Res-DT**.

References.

[S1] F. A. Al-Saif, J. Y. Al-Humaidi, D. N. Binjawhar and M. S. Refat, *J. Mol. Struct.*, 2020, **1218**, 128547.

[S2] M. Du, Y. Zhang, Y. Yu, H. Zhao, Y. Guo and Y. Yang, *Anal. Methods*, 2019, **11**, 6053–6061.

[S3] Y. Zhang, M. Yang and M. Ji, *New J. Chem.,* 2020, **44**, 20434.

[S4] W. Luo, J. Li and W. Liu, *Org. Biomol. Chem.*, 2017, **15**, 5846–5850.

[S5] Q. Xia, S. Feng, D. Liu and G. Feng, *Sens. Actuator B-Chem*, 2018, **258**, 98–104.

[S6] M. Kumar, N. Kumar and V. Bhalla, *RSC Adv.*, 2013, **3**, 1097–1102.

[S7] J. Zhou, S. Xu, Z. Yu, X. Ye, X. Dong and W. Zhao, *Dyes Pigment.*, 2019, **170**, 107656.

[S8] T. Chen, T. Wei, Z. Zhang, Y. Chen, J. Qiang, F. Wang and X. Chen, *Dyes Pigment.*, 2017, **140**, 392–398.

[S9] W. Liu, J. Jiang, C. Chen, X. Tang, J. Shi, P. Zhang, K. Zhang, Z. Li, W. Dou, L. Yang and W. Liu, *Inorg. Chem.*, 2014, **53**, 12590–12594.

[S10] X. Li, H. Huang, Y. Zhu, H. Zhao and Z. Wang, *RSC Adv.*, 2015, **5**, 105810–105813.

[S11] A. Higashi, N. Kishikawa, K. Ohyama and N. Kuroda, *Tetrahedron Lett.,* 2017, **58**, 2774–2778.

[S12] M. E. Jun and K. H. Ahn, *Org. Lett.,* 2010, **12**, 2790–2793.

[S13] M. G. Choi, J.-Y. Seo, E. J. Cho, S.-K. Chang, *J. Photochem. Photobiol. A-Chem*., 2022, **429**, 113920.

[S14] A. Ghosh, S. Nandi, A. Sengupta, A. Chattopadhyay, S. Lohar and D. Das, *Inorg. Chim. Acta,* 2015, **436**, 52–56.
